# Agreement of PROMIS Preference (PROPr) scores generated from the PROMIS-29 + 2 and the PROMIS-16

**DOI:** 10.1007/s11136-024-03827-5

**Published:** 2024-11-07

**Authors:** Janel Hanmer, Chengbo Zeng, Amy M. Cizik, Jason H. Raad, Joel Tsevat, Anthony Rodriguez, Ron D. Hays, Maria Orlando Edelen

**Affiliations:** 1 Division of General Internal Medicine, University of Pittsburgh, Pittsburgh, PA USA; 2 Department of Surgery, Brigham and Women’s Hospital, Patient Reported Outcomes, Value and Experience (PROVE) Center, Boston, MA USA; 3 Department of Orthopaedics, University of Utah, School of Medicine, Salt Lake City, UT USA; 4 Department of Medicine and ReACH Center, Long School of Medicine, University of Texas Health Science Center at San Antonio, San Antonio, TX USA; 5 RAND Corporation, Behavioral and Policy Sciences, 20 Park Plaza #910, Boston, MA USA; 6 UCLA Department of Medicine, Division of General Internal Medicine and Health Services Research, Los Angeles, CA USA

**Keywords:** Health utility, Preference-based measure, HRQOL, Health-related quality of life, Cost-effectiveness analysis, Validation study

## Abstract

**Purpose:**

Preference-based summary scores are used to quantify values, differences, and changes in health-related quality of life (HRQoL) that can be used for cost-effectiveness analyses. The PROMIS-Preference (PROPr) measure is a preference-based summary score comprised of 7 PROMIS domains. The PROMIS-16 is a new PROMIS profile instrument. We evaluated the measurement properties of PROPr generated from the widely used PROMIS-29 + 2 compared with the PROMIS-16.

**Methods:**

We performed a secondary analysis of data from an online survey of the general US population, with a longitudinal subsample who reported back pain. The survey included both the PROMIS-16 and the PROMIS-29 + 2 profiles. PROPr scores were calculated from each profile and compared by the distribution of scores, overall mean scores, product-moment correlations with pain measure scores (Oswestry Disability Index, Roland-Morris Disability Questionnaire, Pain Intensity, Interference with Enjoyment of Life, Interference with General Activity Scale, and Graded Chronic Pain Scale), and difference in mean scores in subgroups with 13 chronic health conditions (Cohen’s d).

**Results:**

Of the 4,115 participants in the baseline survey, 1,533 with any reported back pain were invited for the 6-month follow-up survey and 1,256 completed it. At baseline, the overall mean (SD) PROPr score was 0.532 (0.240) from PROMIS-16 and 0.535 (0.250) from PROMIS 29 + 2. At both time points, the correlations of PROPr scores with physical and mental health summary scores from the PROMIS-29 and 4 pain scales were within 0.01 between profiles. Using subgroups with chronic health conditions and comparing between profiles, Cohen’s d estimates of the difference in effect size were small (< 0.2).

**Conclusion:**

PROPr scores from the 16-item PROMIS profile measure are similar to PROPr scores from the longer PROMIS-29 + 2.

**Supplementary Information:**

The online version contains supplementary material available at 10.1007/s11136-024-03827-5.

## Plain Language summary

The patient-reported outcomes measurement information system (PROMIS^®^) has widely used off-the-shelf profile instruments for adults that yield 8 health-related quality of life domain-specific scores (physical function, ability to participate in social roles and activities, anxiety, depression, sleep disturbance, pain interference, cognitive function - abilities, and fatigue). The shortest profile had been 29 questions. Recently, a 16-item profile (PROMIS-16) was developed to be minimally burdensome and clinically useful. In this report, we test whether an overall summary score of health-related quality of life (named PROPr) scored from the PROMIS-16 has good agreement with scores from the PROMIS-29. We find that the PROPr scores from each profile are essentially the same. These findings support the use of the shorter PROMIS-16 when desired.

**Supplementary Information:**

The online version contains supplementary material available at 10.1007/s11136-024-03827-5.

## Introduction

Patient-reported Outcome Measures (PROMs) are essential for capturing the patient’s perspective. The Patient Reported Outcomes Measurement Information System (PROMIS) developed disease-agnostic, domain-specific PROMs using standardized development and validation methods [1]. PROMIS domain measures are psychometric measures designed to measure the level of a domain (e.g., fatigue, pain interference, physical function) and the PROMIS family currently contains over 80 domains for adults [2]. PROMIS measures are constructed using item response theory (IRT) [3]. IRT provides administration flexibility (e.g., depression can be measured using a standard 4-item short form, standard 8-item short form, computer adaptive test, or custom short form) where scores from each type of administration can be directly compared [4]. For ease of use, several off-the-shelf profiles have been developed for PROMIS that collect information on 7 or 8 domains of health-related quality of life (HRQoL). Previously, the shortest adult PROMIS Profile contained 29 items [5]. We recently developed a 16-item PROMIS Profile and have reported some of its measurement properties [6].

Summary scores that combine multiple domains facilitate comparisons across clinical populations or interventions. The PROMIS domains within the PROMIS profiles have been combined into mental and physical summary scores, with population mean scores of 50 and standard deviations of 10 [7]. Multiple domains can also be combined using preference-based techniques. When a preference-based score is anchored by “dead” at 0 and “full health” at 1.0, the scores are appropriate for estimating quality-adjusted life years (QALYs) for use in decision analyses and cost-effectiveness analyses (CEA) [8–10]. The PROMIS-Preference (PROPr) score is a preference-based score that combines information from 7 PROMIS domains included in a modified version of the 29-item PROMIS profile known as the PROMIS-29 + 2 [11–13].

PROPr was constructed using the input of community members, experts in IRT-based health profile measurement, and experts in preference-based measurement [14]. The final scoring algorithm was estimated using data from a large sample of the US population using multi-attribute utility theory [8, 11]. PROPr is the first scoring system to link single-attribute utility functions to health domains measured by IRT. Because of this unique linking, PROPr gains the advantages of an IRT-based descriptive system including the ability to collect domain information using standard short forms, custom short forms, or computer adaptive testing. PROPr has been shown to correlate with and have similar condition impact estimates to other preference-based scoring systems (i.e., the EQ-5D and Health Utilities Index). However, PROPr has a much lower absolute score than these other systems because the best possible health state described in PROPr is qualitatively much better than those described in these other measures. This has reduced ceiling effects in the general population and mean scores are much lower with PROPr than with these other measures [13, 37]. The PROMIS-29 and PROMIS-29 + 2 Profiles are widely used in research but may be too burdensome for some clinical and research scenarios. To address this need, an ultra-short PROMIS Profile with 16 items was recently developed [6]. The agreement of scores from different PROMIS Profiles is important for comparing studies using different profiles. Agreement in the measured differences and changes are important for CEA studies that have cost-per-QALY thresholds (e.g. an incremental cost-effectiveness ratio of $100,000/QALY) as non-equivalent measures may influence funding decisions [15, 16]. Here, we examine the agreement between PROPr scores generated from the PROMIS-16 and the PROMIS-29 + 2.

## Methods

### Participants

This is a secondary analysis of data from a general US population probability-based sample. Participants were recruited from KnowledgePanel in September and October 2022 [17]. KnowledgePanel is a high-quality, probability-based panel whose members are recruited through an address-based sample method utilizing the most recent delivery sequence file of the US Postal Service. A random sample of 7,224 from the approximately 55,000 KnowledgePanel members were offered the opportunity to participate in the survey [18, 19]. The KnowledgePanel conducted several quality control measures, and the research team included 2 fake conditions within a list of chronic health conditions to identify and exclude careless or insincere respondents [20]. Of those, 4,149 participants agreed to participate but 19 were excluded because of endorsing one of the two fake conditions (“Syndomitis” and “Checkalism”) [20]. Of the remaining 4,130 baseline participants, those experiencing back pain (*n* = 1,533) were selected for a follow-up survey. A total of 277 did not complete the 6-month follow-up survey, leaving 1,256 participants in the 6-month follow-up analysis sample (Supplemental Figure S1).

The study protocol was reviewed and approved by the research team’s institutional review board (RAND Human Subjects Research Committee FWA00003425; IRB00000051). The data set analyzed for this study is publicly available from the ICPSR database repository number openicpsr-198,049.

### Measures

Participant information: At baseline, participants were asked demographic questions and whether they had any chronic conditions including hypertension, high cholesterol, coronary heart disease, angina, heart attack, stroke, asthma, cancer, diabetes, chronic obstructive pulmonary disease, arthritis or rheumatoid arthritis, anxiety disorder, depression, chronic allergies, back pain, chronic back pain, sciatica, neck pain, trouble seeing, dermatitis, stomach trouble, trouble hearing, trouble sleeping, and 2 fake conditions (“Syndomitis” and “Checkalism”). At 6-month follow-up, participants were asked again if they had hypertension, anxiety, and depression.

PROMIS items: As part of the larger study, participants were asked 4 to 8 items from 8 of the PROMIS domain item banks for a total of 50 PROMIS items. Participants answered all items from a domain (e.g., 8 items from the PROMIS Physical Function item bank) before answering the next domain. The selected items included all items in the PROMIS-29 + 2 and PROMIS-16 described below as well as 14 additional items. The PROMIS-29 + 2 and the PROMIS-16 share 11 items.

PROMIS-29 + 2: The PROMIS-29 + 2 Profile evaluates 8 health domains: physical function, ability to participate in social roles and activities, anxiety, depression, sleep disturbance, pain interference, and fatigue with 4 items per domain; cognitive function – abilities with 2 items; and pain intensity with a single item [5]. The domains were scored using IRT-based T-scores from standard PROMIS documentation. T-scores are designed such that 50 is the population mean with a standard deviation of 10. Higher values indicate more of the concept being measured (i.e., higher scores indicate better HRQoL in functioning domains and higher scores indicate worse HRQoL in symptom domains). Physical health and mental health summary scores were also calculated from the PROMIS-29 + 2 [7]. Participants were asked to complete the PROMIS-29 + 2 at baseline and 6-month follow-up.

PROMIS-16: The PROMIS-16 Profile evaluates 8 health domains: physical function, ability to participate in social roles and activities, anxiety, depression, sleep disturbance, pain interference, cognitive function –abilities, and fatigue, with 2 items per domain. We generate IRT-based T-scores for each domain following PROMIS conventions as described in Edelen et al. [6]. Participants were asked the PROMIS-16 items at baseline and 6-month follow-up.

PROPr: The PROPr score is calculated from 7 PROMIS domain scores: cognitive function – abilities, depression, fatigue, pain interference, physical function, sleep disturbance, and ability to participate in social roles [14]. The PROPr scoring algorithm is linked directly to the PROMIS domain T-scores, rather than to individual items, allowing the domain scores to be collected by different administration methods (e.g., computer adaptive test, 4-item short form, 2-item short form). The PROPr scoring algorithm was developed from standard gamble valuations from a US sample of 943 adults. Possible PROPr scores range from − 0.022 to 1.0 with dead anchored at 0 and full health anchored at 1.0 [11]. PROPr scores were calculated using each profile, hereafter referred to as the “PROPr_16_” and “PROPr_29 + 2_.”

Pain-specific measures: To validate PROPr scores derived from the PROMIS-16, we included 4 pain measures, the: (1) Oswestry Disability Index (ODI) [21, 22], (2) Roland-Morris Disability Questionnaire (RMDQ) [23], (3) Pain Intensity, Interference with Enjoyment of Life, Interference with General Activity Scale (PEG) [24], and (4) Graded Chronic Pain Scale (GCPS) [25]. The ODI measures pain interference and functional disability by using 10 items that assess pain intensity, personal care, lifting, walking, sitting, standing, sleeping, sex life, social life, and traveling. Each item is rated on a 0 to 5 scale, yielding a total sum score ranging from 0 to 50. We transformed the sum score to a percentage scale from 0 to 100, categorizing disability into minimal (0–20%), moderate (21–40%), severe (41–60%), disabling (61–80%), and bedridden or functional impairment (81– 100%). The RMDQ, with a range from 0 to 24, evaluates if back pain has an impact on 24 daily activities, with higher scores indicating greater impact. The PEG uses a single item to assess pain intensity and 2 items to assess interference with enjoyment of life and general activities. Each item is rated on a 0 to 10 scale and the total score, ranging from 0 to 10, is the average of these 3 item scores. Lastly, the GCPS has 3 pain intensity items and 4 disability items. Following previous studies, we scored GCPS and classified the severity as (1) no pain, (2) low disability – low intensity, (3) low disability – high intensity, (4) high disability – moderately limiting, and (5) high disability – severely limiting [26]. Participants were asked to complete all 4 pain measures at baseline and 6-month follow-up.

### Analysis

First, we examined the difference in the score distributions between PROPr_16_ and PROPr_29 + 2_ using the Kolmogorov-Smirnov test and score correlations using product-moment correlations [27]. Second, we calculated mean scores for the overall sample and subsets with different health conditions and calculated the standardized mean difference (Cohen’s d) between group mean estimates from PROPr_16_ and PROPr_29 + 2_; a Cohen’s d statistic less than 0.2 indicates a trivial difference [28]. Third, PROPr score correlations with other pain and disability measures (GCPS, ODI, RMDQ, and PEG) and PROMIS summary scores were calculated by using product-moment correlations [27]. Fourth, the impact of the respondent’s health condition on the PROPr_16_ and PROP_29 + 2_ scores were estimated in linear multivariable regression analyses controlling for age and sex. The regression coefficient for the health condition is its impact estimate. Fifth, we created a Bland-Altman plot with the average of the PROPr_16_ and PROPr_29 + 2_ to help identify any systematic differences between the PROPr_16_ and PROP_29 + 2_ scores [29]. The 95% upper and lower limits of agreement (bias) are estimated using: mean ± SD (mean difference) * 1.96. Scatter bias is present when the amount of disagreement varies by the average of the two estimates. Finally, participants were categorized into 3 groups based on the change in pain severity from baseline to the 6-month follow-up: “decreased,” “no change,” or “increased” that was measured using ODI and GCPS [22, 25]. For each group, we calculated the change of PROPr_16_ and PROPr_29 + 2_ scores. We then examined whether the differences between the 2 profiles in these changes were statistically significant by using the Wilcoxon rank sum test to account for the non-normality of the change score distributions.

A 2-sided p-value less than 0.05 was considered statistically significant for all statistical analyses. Analyses were performed in SAS version 9.4.

## Results

A description of the participant sample has been provided in a previous publication and is included in Supplemental Table 1. Over half of the baseline survey participants (59%) were under age 60, 40% had a bachelor’s degree, 70% were white, non-Hispanic, and 50% were female. The most reported chronic conditions at baseline were allergies (45%), hypertension (38%), and high cholesterol (38%).

Of the 1,533 people in the baseline survey who had back pain, 1,256 (82%) completed the 6-month follow-up survey. The demographic characteristics of the study sample at the 6-month follow-up were generally consistent with those at baseline. Allergies (58%), hypertension (47%), and high cholesterol (47%) were the most common chronic conditions.

**Fig. 1:**
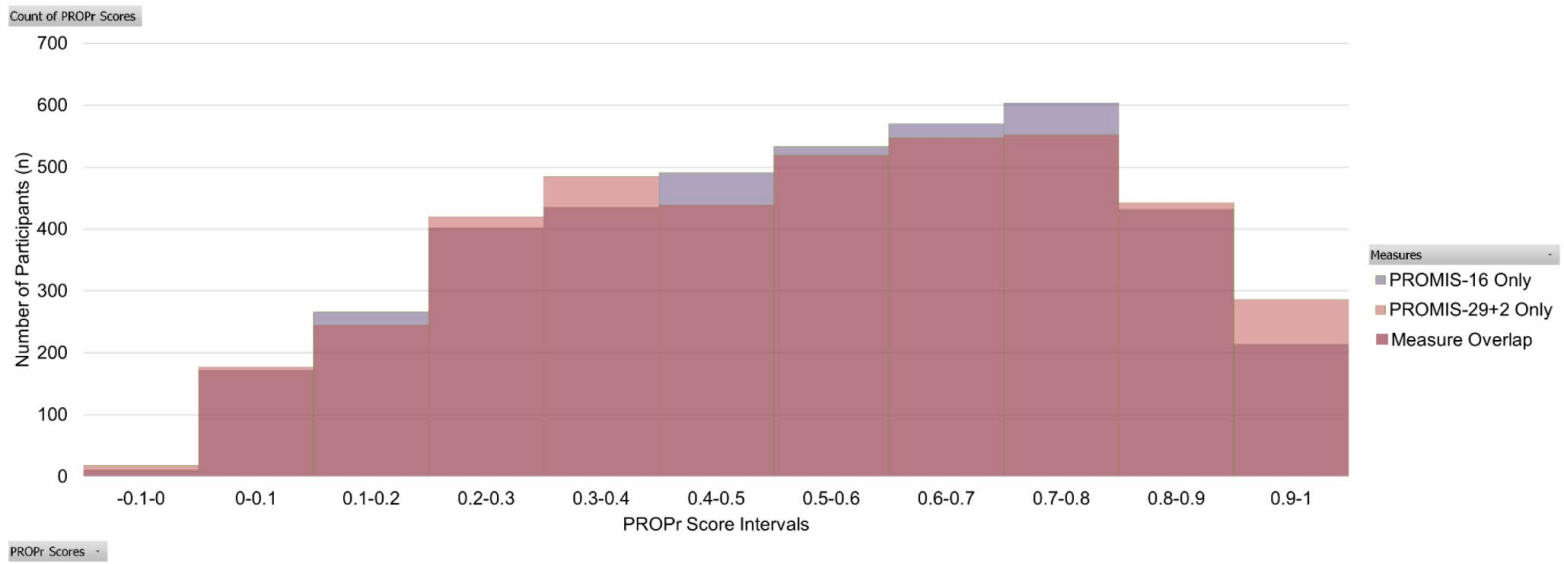
Distribution of PROPr_16_ and PROPr_29 + 2_ scores at Baseline

In the overall baseline sample, the mean (SD) PROPr_16_ score was 0.532 (0.241) and for the PROPr_29 + 2_ it was 0.535 (0.251; standardized mean difference: 0.012; Table 1; Fig. 1). In the 6-month follow up of participants reporting back pain at baseline, the mean (SD) PROPr_16_ score was 0.429 (0.229) vs. 0.423 (0.233) on the PROPr_29 + 2_ (standardized mean difference: 0.028; Supplemental Figure S2). The 2 versions were correlated at 0.93 at baseline and 0.95 at 6-month follow-up.

PROPr_16_ and PROPr_29 + 2_ had small but significantly different distributions at baseline (*p* < 0.001) by the Kolmogorov-Smirnov test. Specifically, slightly more participants had PROPr_16_ scores than PROPr_29 + 2_ scores in the range of 0.4 to 0.8. Conversely, the number of participants with PROPr_29 + 2_ scores in the ranges of 0.2 to 0.4 and 0.8 to 1 was slightly higher than those with PROPr_16_ scores in these intervals. At the 6-month follow-up, the distributions of PROPr_16_ and PROPr_29 + 2_ were not significantly different by the Kolmogorov-Smirnov test (*p* = 0.526).

**Table 1:** Standardized effect sizes (Cohen’s d) of mean PROPr_16_ vs. mean PROPr_29 + 2_ scores at baseline and the 6-month follow-up

Groups	Baseline (*N* = 4130)			6-month follow-up (*N* = 1256)		
	PROPr_16_	PROPr_29 + 2_	Cohen’s d	PROPr_16_	PROPr_29 + 2_	Cohen’s d
Overall	0.532 (0.241)	0.535 (0.251)	-0.012	0.429 (0.229)	0.423 (0.234)	0.028
Hypertension	0.483 (0.241)	0.486 (0.251)	-0.011	0.405 (0.226)	0.393 (0.231)	0.05
Cholesterol	0.500 (0.242)	0.503 (0.253)	-0.01	0.411 (0.228)	0.403 (0.232)	0.032
Heart disease	0.431 (0.246)	0.427 (0.260)	0.015	0.393 (0.222)	0.387 (0.236)	0.026
Angina	0.349 (0.239)	0.342 (0.252)	0.029	0.298 (0.199)	0.293 (0.207)	0.025
Heart attack	0.414 (0.245)	0.408 (0.253)	0.022	0.361 (0.221)	0.357 (0.246)	0.019
Stroke	0.393 (0.239)	0.395 (0.252)	-0.006	0.405 (0.202)	0.378 (0.217)	0.127
Asthma	0.435 (0.248)	0.437 (0.255)	-0.006	0.350 (0.235)	0.341 (0.237)	0.036
Cancer	0.493 (0.227)	0.500 (0.239)	-0.031	0.436 (0.210)	0.427 (0.222)	0.045
Diabetes	0.434 (0.242)	0.432 (0.250)	0.01	0.345 (0.207)	0.340 (0.211)	0.028
COPD	0.370 (0.248)	0.365 (0.253)	0.018	0.306 (0.217)	0.301 (0.217)	0.02
Arthritis	0.431 (0.230)	0.435 (0.240)	-0.017	0.369 (0.215)	0.361 (0.219)	0.037
Anxiety	0.360 (0.226)	0.360 (0.230)	0.001	0.299 (0.212)	0.295 (0.217)	0.018
Depression	0.340 (0.216)	0.341 (0.220)	-0.003	0.283 (0.201)	0.281 (0.206)	0.011

Cohen’s d effect sizes are usually interpreted as very small (< 0.01), small (< 0.20), medium (< 0.50), large (< 0.80) or very large ( > = 0.80)

Table 1 includes mean scores for the overall sample and subsets with different health conditions. The table also includes the standardized mean difference (Cohen’s d) between group means from PROPr_16_ and PROPr_29 + 2_. All Cohen’s d statistics were less than 0.2 indicating trivial differences [28].

The PROPr scores from PROMIS-16 and PROMIS-29 + 2 were strongly associated with the GCPS, ODI, PEG, RMDQ, and PROMIS physical and mental health summary scores (Table 2) [27]. The direction and strength of the product-moment correlations were virtually identical between PROPr_16_ and PROPr_29 + 2_ scores.

**Table 2:** Product-moment correlations of PROPr with four pain scales, PROMIS Mental Health, and PROMIS Physical Health at the baseline and 6-month follow-up

Measure	ODI	RMDQ	PEG	GCPS	PROMIS-29 Physical health Summary Score	PROMIS-29 Mental health Summary Score
**Baseline (** ***N*** ** = 4130)**						
PROPr_16_	-0.67	-0.62	-0.69	-0.59	0.74	0.87
PROPr_29 + 2_	-0.68	-0.62	-0.70	-0.59	0.74	0.86
**6-month (** ***N*** ** = 1256 reporting any back pain at baseline)**						
PROPr_16_	-0.69	-0.65	-0.72	-0.59	0.78	0.90
PROPr_29 + 2_	-0.70	-0.65	-0.72	-0.59	0.80	0.90

ODI: Oswestry Disability Index

PEG: Pain Intensity, Enjoyment of life, and interference with General activity

GCPS: Graded Chronic Pain Scale

**Fig. 2:**
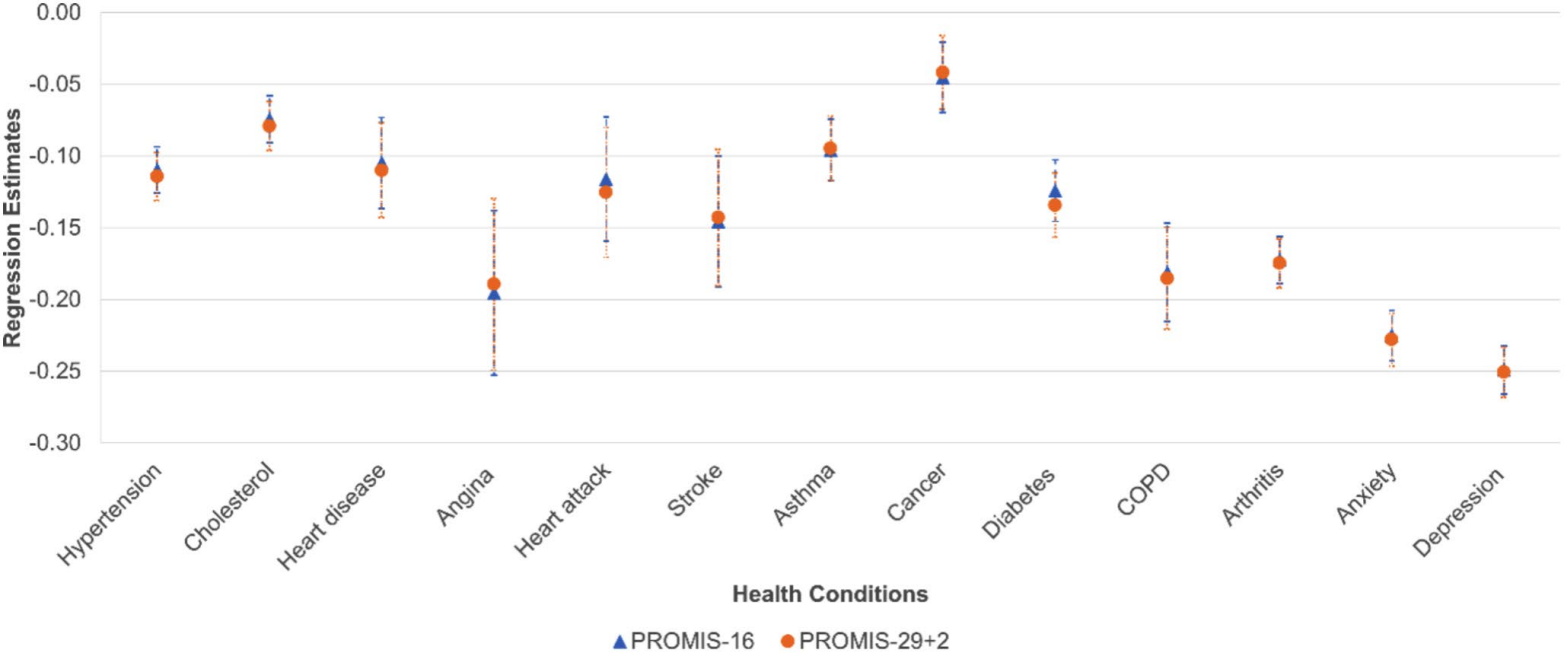
Health condition impact estimates from regression analyses of PROPr scores at baseline. Note Age and gender were adjusted as covariates. Error bars were the 95% confidence intervals (CIs) for the point estimates

Regression analyses of PROPr_16_ and PROPr_29 + 2_ scores, as predicted by each health condition at baseline, showed that the impact estimates for the PROPr scores generated by PROMIS-16 and PROMIS-29 + 2 are virtually identical, with overlapping confidence intervals (Fig. 2). Regression analyses using data at 6-month follow-up yielded consistent findings and are included in the supplementary materials (Supplement Figure S3).

**Fig. 3:**
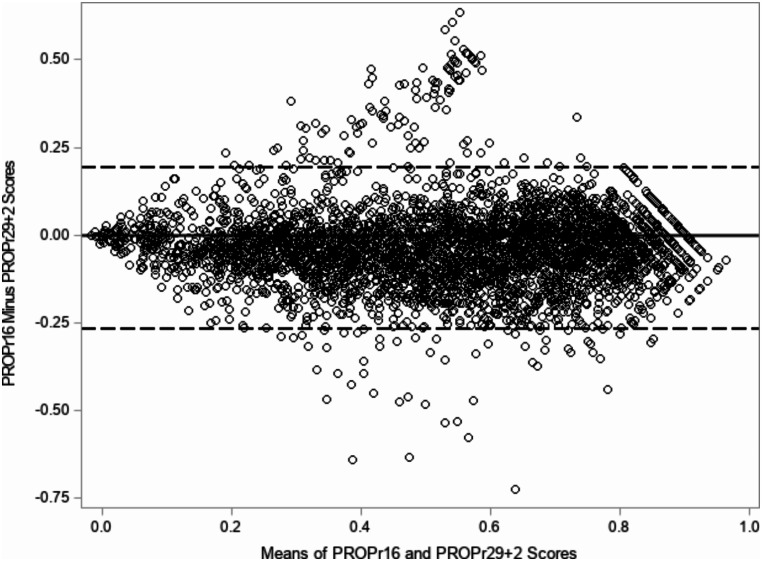
*Bland-Altman plot for the PROPr*_*16*_*and PROPr*_*29 + 2*_*scores at baseline.* Note Solid line indicates the mean difference between PROPr_16_ and PROPr_29 + 2_; Dash lines indicate the 95% upper and lower limits of agreement

The Bland-Altman plot shows scatter bias for the difference between PROPr_16_ and PROPr_29 + 2_ scores, with PROMIS-16 slightly overestimating the PROMIS-29 + 2 at the upper (better PROPr score) end of the distribution (Fig. 3).

**Table 3:** Longitudinal change in PROPr scores according to the ODI and GCPS Change in Pain Severity in Back Pain Cohort (*n* = 1256) at baseline and 6-month follow-up

Change in Measure	Mean difference (95%CI)		*p*-value
	Change in PROPr_16_	Change in PROPr_29 + 2_	
**ODI pain severity**			
Decreased	-0.051 (-0.071, -0.030)	-0.053 (-0.074, -0.033)	0.665
No Change	0.001 (-0.008, 0.010)	-0.005 (-0.014, 0.004)	0.388
Increased	0.069 (0.048, 0.090)	0.068 (0.046, 0.090)	0.913
**GCPS pain severity**			
Decreased	-0.046 (-0.066, -0.026)	-0.049 (-0.070, -0.028)	0.857
No Change	0.001 (-0.009, 0.011)	-0.004 (-0.014, 0.007)	0.415
Increased	0.063 (0.037, 0.089)	0.060 (0.036, 0.084)	0.776

Both PROPr_16_ and PROPr_29 + 2_ changed significantly by the change in pain severity as defined by the ODI from baseline to 6 months, with a mean PROPr_16_ difference of -0.051 (95% CI: -0.071, -0.030) for those whose pain severity decreased and a mean difference of 0.069 (95%CI: 0.048, 0.090) for those whose pain severity increased (Table 3). For participants without a change in pain severity, the mean difference in the PROPr_16_ and PROPr_29 + 2_ scores was not significant. Across the 3 levels of pain severity, the differences in changes between PROPr_16_ and PROPr_29 + 2_ from baseline to 6 months were not significant. Similar results were found when using the GCPS to define levels of pain severity and disability.

## Discussion

The PROMIS-16 was developed to serve as a brief PROMIS profile that could be administered in clinical and research scenarios where longer profile instruments would be considered too burdensome [6]. Preference-based summary scores of health, like PROPr, are a useful tool for comparing health states by how the population values them. They are a necessary input to CEAs that utilize QALYs [15]. Because of the way they are used, differences and changes in scores are more important than absolute scores [12]. In this study, we examined if PROPr scores from the new PROMIS-16 Profile agree with PROPr scores derived from the PROMIS-29 + 2 Profile and found them to be broadly commensurate. Specifically, mean PROPr scores generated from the two profiles were within 0.006 of each other and correlations with other HRQoL scores were essentially identical across profile versions. We also found that mean scores by health condition were similar and that health condition impact estimates, when adjusted for age and sex, were similar between PROPr_16_ and PROPr_29 + 2_ (mean difference 0.00, range − 0.007–0.007). In groups that reported improvement, no change, or no improvement on the ODI or GCPS, changes in scores were within 0.004 between PROPr_16_ and PROPr_29 + 2_.

Of note, there was a difference in the distribution of PROPr_16_ and PROPr_29 + 2_ in the baseline data, in which PROPr_16_ had more scores clustering in the 0.4 to 0.8 range and PROPr_29 + 2_ had more scores at the tails of the distributions (0.2 to 0.4 and 0.8 to 1.0). This result may be due to the somewhat reduced range of domain scores from the PROMIS-16 when compared with the PROMIS-29 [5, 6, 30], which in turn would restrict the possible scores of PROPr_16_ when compared with PROPr_29 + 2_. These distributional differences did not impact overall mean scores and were not observed in the follow-up data.

Similarities in PROPr scores from the PROMIS-16 and PROMIS-29 + 2 is expected because the scoring algorithm for PROPr is linked to the underlying PROMIS domain scores rather than to individual PROMIS items. PROMIS-16 and PROMIS-29 + 2 both use IRT-based scoring of these underlying domains. This allows PROPr scoring to use the unique information each profile provides. The absolute PROPr scores found in this report (e.g., an overall mean of 0.53 at baseline in the general population sample) are consistent with other general population reports. PROPr’s absolute score is lower than preference-based scores, such as the EQ-5D and Health Utilities Index, because its descriptive system covers a wider range of mild health states.

Despite their usefulness in CEA, measures that generate preference based HRQoL scores are usually not collected routinely in clinical care. This is both because routine collection of PROMs is difficult and because a preference-based summary score of health is not intended to guide the care of an individual patient [31–33]. An ultra-short profile such as the PROMIS-16 allows clinically meaningful information (such as domain scores for depression, fatigue, and pain) to be collected for managing individual patients. Depending on administration conditions, it takes 4.6 to 10 s per PROMIS item. The PROMIS-16 should save between 1 min 9 s and 2 min 30 s of data collection time compared to the PROMIS-29 + 2 [34, 35]. Then these same data can be aggregated into a preference-based score for comparisons across populations (such as across different patient characteristics) or for more formal comparisons (such as CEA) and can be useful for health systems for population health management [36].

Limitations of this study include using an online panel in which longitudinal data were only collected from participants who reported back pain at baseline, so these results may not be generalizable to other populations. The drop out in the longitudinal sample was 18% and it is unknown what factors may be associated with differential drop out rates and the measures used in this study. Also, the content overlap of the legacy pain measures to PROMIS profiles is high, but there may be incomplete overlap (e.g., the ODI has items about sexual function). The health conditions evaluated in this study were self-reported, but data were cleaned to exclude people who endorsed either of the two fake conditions [20]. This study is also limited because it does not compare PROPr scores from the PROMIS-16 to other PROMIS collection methods, such as PROMIS-29-CAT or PROMIS-43, nor did it compare them with other preference-based measures such as the EQ-5D or Health Utilities Index [37–39]. This study assumes that the PROMIS-16 and PROMIS-29 + 2 items extracted from a larger study in which these items were intermingled and administered with other PROMIS items are consistent with responses expected if each form were administered separately. There is a small amount of evidence that order effects in PROMIS measures are small [40] and that the IRT parameter invariance assumption is appropriate [41]. However, future work could test this assumption further by administering each of these specific forms separately. Those limitations notwithstanding, this report has provided evidence that PROPr scores from the PROMIS-16 and the longer PROMIS-29 + 2 profiles are essentially equivalent to each other in both the general US population and a population with back pain. These results suggest that PROPr scores from the PROMIS-16 and PROMIS-29 + 2 could be considered interchangeable for monitoring the health of populations, comparing HRQoL across groups, evaluating overall HRQoL change over time within groups, and CEA.

## Electronic supplementary material

Below is the link to the electronic supplementary material.

Supplementary Material 1

Supplementary Material 2

Supplementary Material 3

Supplementary Material 4

## Data Availability

The data set analyzed for this study is publicly available from the ICPSR database repository number openicpsr-198,049.
