# Supplementary figures and images for: Agreement of PROMIS Preference (PROPr) scores generated from the PROMIS-29 + 2 and the PROMIS-16
Source: Qual Life Res. 2024 Nov 7;34(1):43–51. doi: 10.1007/s11136-024-03827-5 (PMC11802291; doi:10.1007/s11136-024-03827-5)

**Fig. S1** Study flowchart of the KnowledgePanel dataset


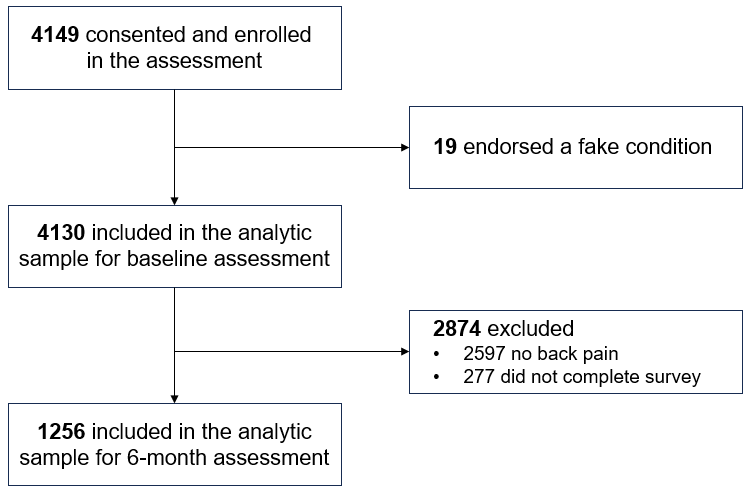

Supplement: Supplementary file 1 — Supplementary Material 1 [file 11136_2024_3827_MOESM1_ESM.docx]

**Fig. S2** Distribution of PROPr16 and PROPr29+2 Scores at Month 6


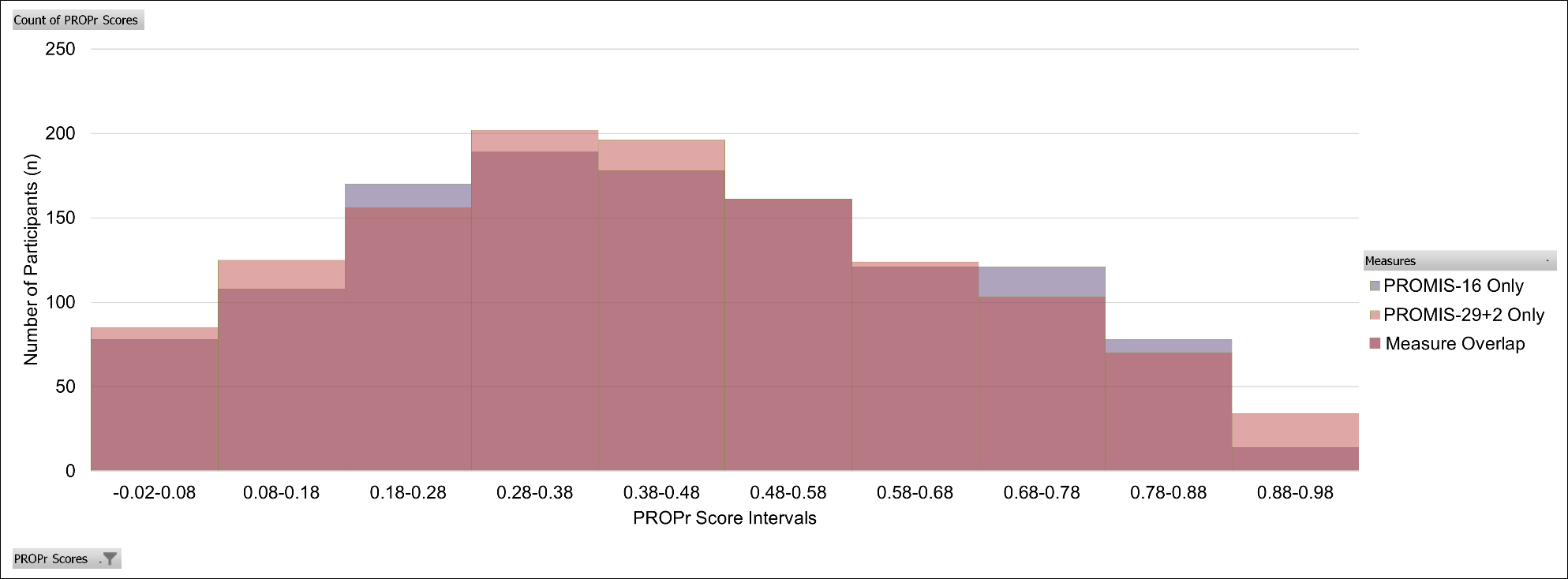

Supplement: Supplementary file 2 — Supplementary Material 2 [file 11136_2024_3827_MOESM2_ESM.docx]
