# Supplementary material for: Agreement of PROMIS Preference (PROPr) scores generated from the PROMIS-29 + 2 and the PROMIS-16
Source: Qual Life Res. 2024 Nov 7;34(1):43–51. doi: 10.1007/s11136-024-03827-5 (PMC11802291; doi:10.1007/s11136-024-03827-5)
Supplement: Supplementary file 3 — Supplementary Material 3 [file 11136_2024_3827_MOESM3_ESM.docx]

**Fig. S3** Health condition impact estimates from regression analyses of PROPr scores at six-month follow-up*


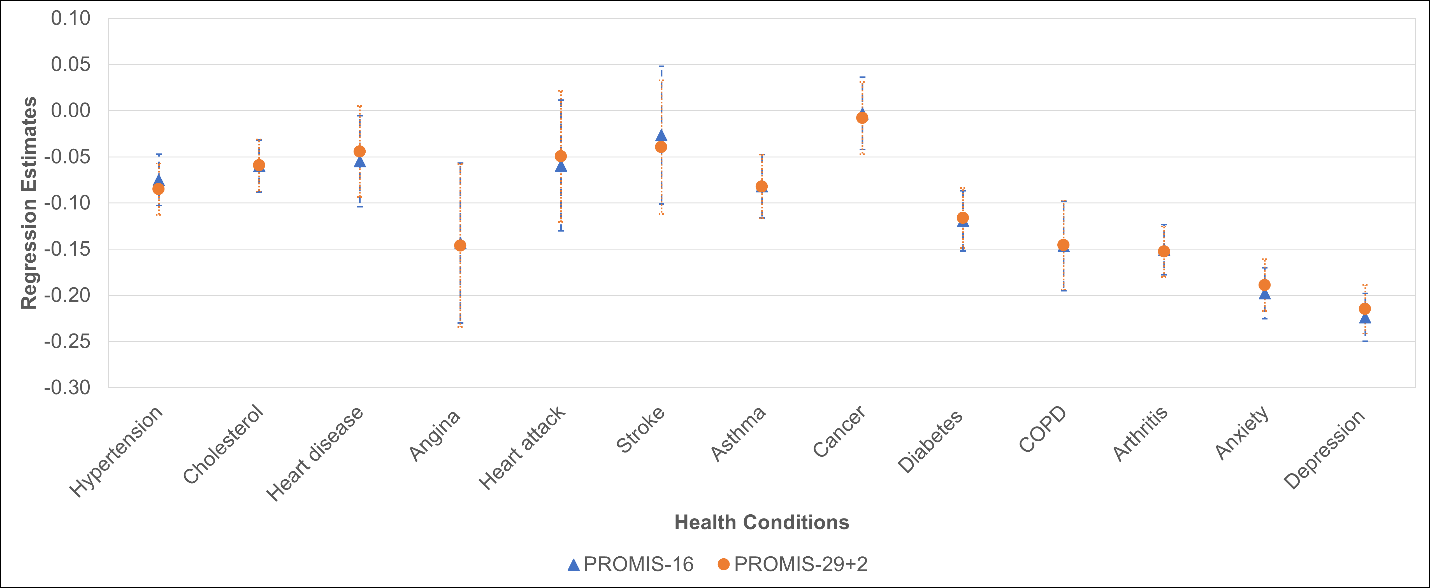


*Hypertension, anxiety, and depression were re-collected at six-month follow-up. All other conditions were reported at baseline.

Age and gender were adjusted as covariates.

Error bars were the 95% CIs of the point estimates.
