# Supplementary material for: Agreement of PROMIS Preference (PROPr) scores generated from the PROMIS-29 + 2 and the PROMIS-16
Source: Qual Life Res. 2024 Nov 7;34(1):43–51. doi: 10.1007/s11136-024-03827-5 (PMC11802291; doi:10.1007/s11136-024-03827-5)
Supplement: Supplementary file 4 — Supplementary Material 4 [file 11136_2024_3827_MOESM4_ESM.docx]

**Table S1.** Demographic characteristics of participants in Knowledge Panel sample over time

| **Characteristics** | **Baseline (N=4130)** | **6-month (N=1256)** |
| --- | --- | --- |
| **Age** |  |  |
| 18 to 29 years of age | 560 (13.6) | 130 (10.4) |
| 30 to 44 years of age | 953 (23.1) | 251 (20.0) |
| 45 to 60 years of age | 911 (22.1) | 289 (23.0) |
| older than 60 years of age | 1706 (41.3) | 586 (46.7) |
| **Race** |  |  |
| White, non-Hispanic | 2887 (69.9) | 932 (74.2) |
| Black, non-Hispanic | 414 (10.0) | 98 (7.8) |
| Other, non-Hispanic | 195 (4.7) | 47 (3.7) |
| Multiracial, non-Hispanic | 497 (12.0) | 126 (10.0) |
| Hispanic | 137 (3.3) | 53 (4.2) |
| **Sex** |  |  |
| Female | 2035 (49.5) | 661 (52.7) |
| Male | 2049 (49.8) | 589 (47.0) |
| Transgender | 11 (0.3) | 4 (0.3) |
| Do not identify as female, male, or transgender | 17 (0.4) | 0 (0.0) |
| **Education** |  |  |
| No high school diploma or GED | 279 (6.8) | 87 (6.9) |
| High school graduate or GED | 1097 (26.6) | 354 (28.2) |
| Some college, or Associate’s degree | 1087 (26.3) | 364 (29.0) |
| Bachelor's degree | 909 (22.0) | 245 (19.5) |
| Master’s degree or higher | 758 (18.4) | 206 (16.4) |
| **Income** |  |  |
| Less than $10,000 | 121 (2.9) | 51 (4.1) |
| $10,000 to 49,999 | 1006 (24.4) | 351 (28.0) |
| $50,000 to 99,999 | 1258 (30.5) | 398 (31.7) |
| $100,000 or more | 1745 (42.3) | 456 (36.3) |
| **Health conditions** |  |  |
| Hypertension | 1570 (38.2) | 592 (47.2) |
| High Cholesterol | 1517 (37.6) | 580 (47.2) |
| Coronary heart disease | 237 (5.8) | 97 (7.8) |
| Angina, also called angina pectoris | 65 (1.6) | 27 (2.2) |
| Heart attack | 120 (2.9) | 43 (3.4) |
| Stroke | 106 (2.6) | 41 (3.3) |
| Asthma | 532 (13.0) | 210 (16.8) |
| Cancer or a malignancy of any kind | 417 (10.2) | 164 (13.1) |
| Diabetes | 548 (13.4) | 240 (19.3) |
| Chronic Obstructive Pulmonary Disease, COPD, emphysema, or chronic bronchitis | 191 (4.7) | 96 (7.7) |
| Some form of arthritis, rheumatoid arthritis, gout, lupus, or fibromyalgia | 1208 (29.5) | 573 (45.8) |
| Any type of anxiety disorder | 806 (19.7) | 368 (29.5) |
| Any type of depression | 820 (20.0) | 378 (30.2) |
| Chronic or seasonal allergies or sinus trouble | 1860 (45.3) | 732 (58.4) |
| Back pain | 1533 (37.7) | 977 (78.0) |
| Chronic back pain | 865 (20.9) | 708 (56.5) |
| Sciatica or radiating leg pain | 695 (16.9) | 411 (32.8) |
| Neck pain | 811 (19.7) | 514 (41.2) |
| Trouble seeing, even when wearing glasses or contact lenses | 596 (14.5) | 269 (21.5) |
| Dermatitis or other chronic skin rash | 423 (10.3) | 181 (14.5) |
| Stomach trouble | 630 (15.4) | 314 (25.1) |
| Trouble hearing, including deafness, in one or both ears | 628 (15.3) | 287 (22.9) |
| Trouble sleeping | 1149 (28.0) | 611 (48.7) |

Note: The size of some cell samples was smaller than the overall sample size because of missing data for one of the variables. Hypotension, depression, anxiety, back pain, neck pain, and trouble sleeping were collected at 6-month. GED: General education diploma
